# A Participant-Derived Xenograft Mouse Model to Decode Autologous Mechanisms of HIV Control and Evaluate Immunotherapies

**DOI:** 10.21769/BioProtoc.5254

**Published:** 2025-04-05

**Authors:** Emma Falling Iversen, Itzayana G. Miller, Ole Søgaard, Ali Danesh, Brad R. Jones

**Affiliations:** 1 Department of Clinical Medicine, Aarhus University, Aarhus, Denmark; 2 Division of Infectious Diseases, Department of Medicine, Weill Cornell Medical College, New York, NY, USA; 3 Department of Microbiology and Immunology, Weill Cornell Graduate School of Medical Sciences, New York, NY, USA; 4 Department of Infectious Diseases, Aarhus University Hospital, Aarhus, Denmark

**Keywords:** HIV, Autologous, Participant-derived, T cells, Immunotherapies, Small animal

## Abstract

Human immunodeficiency virus (HIV) remains a global health challenge with major research efforts being directed toward the unmet needs for a vaccine and a safe and scalable cure. Antiretroviral therapy (ART) suppresses viral replication but does not cure infection and so requires lifelong adherence. HIV-specific CD8+ T-cell responses play a crucial role in long-term HIV control as demonstrated in *elite controllers*, highlighting their potential in HIV cure strategies. Various HIV mouse models—including the human-hematopoietic stem cell (Hu-HSC) mouse, the bone marrow, liver, and thymus (BLT) mouse, and the human peripheral blood leukocyte (Hu-PBL) mouse—have deepened the understanding of HIV dynamics and facilitated the development of therapeutics. We developed the HIV participant-derived xenograft (HIV PDX) mouse model to enable long-term in vivo evaluation of bona fide autologous T-cell mechanisms of HIV control, including the antiviral activity of primary memory CD8+ (mCD8+) T cells taken directly from people with or without HIV, as well as testing potential immunotherapies. Additionally, this model faithfully recapitulates virus escape mutations in response to sustained CD8+ T-cell pressure, enabling the assessment of strategies to curb virus escape. In this model, NSG mice are engrafted with purified memory CD4+ (mCD4+) cells and infected with HIV; then, they receive autologous CD8+ T cells or T-cell products. Key advantages of this model include the minimization of graft-versus-host disease (GvHD), which severely limits peripheral blood mononuclear cell (PBMC) or total CD4-engrafted mice, the ability to evaluate long-term natural donor-specific T-cell responses in vivo, and the lack of use of human fetal tissues required for most humanized mouse models of HIV.

Key features

• Long-term evaluation of bona fide autologous T cells.

• Evaluation of immunomodulating drugs and T-cell products.

• The protocol requires access to a BSL2+ tissue culture room, BSL2+ animal facility, and 6+ weeks to complete.

## Graphical overview


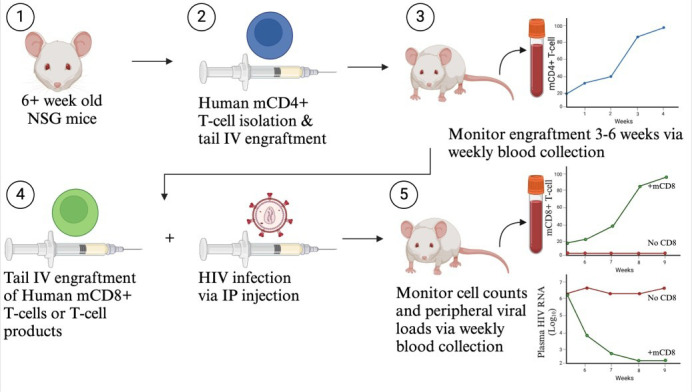


**HIV participant-derived xenograft (HIV PDX) mouse model.** (1) Use >6-week-old NSG mice. (2) Purify human memory CD4+ T cells and engraft via tail intravenous (IV) injection. (3) Monitor mCD4+ T-cell counts for 3–6 weeks until levels exceed 50 mCD4+ T cells/µL. (4) Infect with HIV via intraperitoneal (IP) injection and engraft autologous CD8+ T cells or T-cell products via tail IV injection. (5) Monitor cell counts and plasma viral loads via weekly blood collection.

## Background

Human immunodeficiency virus (HIV) remains a global health challenge with 39.9 million people with HIV (PWH) globally and 1.3 million new infections occurring annually [1]. HIV replication can be suppressed by antiretroviral therapy (ART), allowing PWH to maintain undetectable viral levels. ART prevents both disease progression and viral transmission to sexual partners; however, ART does not cure the infection and thus requires lifelong adherence [2,3]. Major research efforts are being directed toward the unmet needs for a vaccine and a safe and scalable cure. In a minor subset of PWH, termed *elite controllers*, the HIV-specific CD8 T-cell responses play a crucial role in the spontaneous control of viral replication in the absence of ART [4]. Elucidating the mechanisms of HIV control demonstrated by elite controllers may lead to vaccination or immunotherapeutic strategies to engender immune control in the general population of PWH. A mouse model of HIV that recapitulates key features (as well as limitations, such as immune escape) of CD8+ T cell–mediated control would greatly facilitate these efforts.

Murine cells do not support HIV infection or replication. This has led to the development of “humanized mice” where immunodeficient mouse strains are xenografted with human cells and/or tissues to generate CD4+ T cells supporting HIV replication [5–7]. Notable models include i) the human peripheral blood leukocyte (Hu-PBL-SCID) model, generated by injection of human PBMCs into the peritoneal cavity of CB17-severe immunodeficiency (SCID) mice [8], ii) the human hematopoietic stem cell (Hu-HSC) model, generated by sublethal irradiation of newborn NOD SCID IL2r^null^ (NSG) mice and reconstitution with human hematopoietic stem cells (HSCs) derived from fetal liver, cord blood, or bone marrow [9,10], and iii) the bone marrow, liver, and thymus (BLT) model, generated by implantation of NSG mice with fetal liver and thymus tissue and then sublethally irradiated and injected with Hu-HSCs [11,12]. These HIV mouse models have deepened the understanding of HIV dynamics and facilitated the development of therapeutics. However, the need and ethical considerations related to the use of human fetal tissues in addition to the onset of graft-versus-host disease (GvHD) limit these mouse models [7,13].

We developed the HIV participant-derived xenograft (HIV PDX) mouse model to enable long-term in vivo evaluation of bona fide autologous T-cell mechanisms of HIV control, including the antiviral activity of primary memory CD8+ (mCD8+) T cells taken directly from adult PWH or HIV-negative donors. One major advantage of this model is the minimization of GvHD, allowing for experimental timelines greater than one year. This was achieved by excluding naive T cells based on the rationale that these, rather than memory T cells, give rise to GvHD. This model robustly recapitulates virus escape mutations in response to sustained CD8 T-cell pressure, enabling the assessment of strategies to curb virus escape. This model uniquely offers the opportunity to test autologous T cells, engineered T-cell therapies, immunomodulatory agents, and combinations thereof. Specifically, in the HIV PDX model, NSG mice are engrafted intravenously with mCD4+ T cells. Following mCD4+ T-cell expansion, mice are infected with HIV and further receive autologous mCD8+ T cells or T-cell products. If desired, immunomodulating agents or ART can be administered. We have leveraged this model to provide pre-clinical efficacy data on T-cell therapy products prior to initiating a clinical trial (NCT04975698) and to assess a targeted delivery platform for IL-15 [14]. The HIV PDX model thus provides a valuable platform in which to examine the roles of CD8+ T cells in natural HIV control as well as to bridge the development of T cell–focused immunotherapeutics from in vitro studies to clinical HIV trials [14].

## Materials and reagents

**Biological materials**

1. NOD.Cg-*Prkdc^scid^ Il2rg^tm1Wjl^*/SzJ mice (The Jackson Laboratory, strain: 005557, 6–8 weeks of age), hereafter referred to as NSG mice

2. Human embryonic kidney 293T (HEK293T) cells (American Type Culture Collection, catalog number: CRL-3216)

3. TZM-bl cells (JC53bl-13) (NIH AIDS Research and Reference Reagent Program, catalog number: ARP-8129)

4. HIV JR-CSF plasmid (NIH AIDS Research and Reference Reagent Program, catalog number: ARP-2708)

5. Human PBMCs (see ethical considerations)

**Reagents**

1. DMEM (Gibco, catalog number: 11965084)

2. RPMI-1640 (Gibco, catalog number: 11875085)

3. DPBS (Gibco, catalog number: 14190136)

4. EDTA (Fisher Scientific, catalog number: AM9260G)

5. HEPES (Gibco, catalog number: 15630080)

6. L-Glutamine (Gibco, catalog number: 25030081)

7. Penicillin/streptomycin (Gibco, catalog number: 15070063)

8. Fetal bovine serum (FBS) (Gibco, catalog number: 16140071)

9. Teceleukin (recombinant human interleukin-2, IL-2) (Roche, catalog number: 23-6019)

10. PEG-it (System Biosciences, catalog number: LV810A-1)

11. DEAE dextran (Sigma-Aldrich, catalog number: D9885)

12. Cell culture lysis 5× reagent (Promega, catalog number: E153A)

13. Bright-Glo luciferase assay system (Promega, catalog number: E2610)

14. Glo lysis buffer (Promega, catalog number: E2661)

15. EasySep^TM^ Human Memory CD4+ T-Cell Enrichment kit (STEMCELL Technologies, catalog number: 19157)

16. EasySep^TM^ Human Memory CD8+ T-Cell Enrichment kit (STEMCELL Technologies, catalog number: 19159)

17. EasySep^TM^ Human CD8 Positive Selection kit II (STEMCELL Technologies, catalog number: 17853) (optional)

18. UltraComp eBeads^TM^ compensation beads (Life Technologies, catalog number: 01-2222-42)

19. CountBright absolute counting beads (Invitrogen, catalog number: C36950)

20. Viability Marker Live/Dead Fixable aqua Dead Cell Stain kit (Invitrogen, catalog number: L34957) and anti-human antibodies for flow cytometry: PerCP/Cyanine5.5 CD45 (BioLegend, catalog number: 304028), BV785 CD3 (BioLegend, catalog number: 344842), BV421 CD4 (BioLegend, catalog number: 300532), BV711 CD8 (BioLegend, catalog number: 301043), AF700 CD45RO (BioLegend, catalog number: 304218), BV605 CD56 (BioLegend, catalog number: 318334), APC CD14 (BioLegend, catalog number: 367118), AF488 CD16 (BioLegend, catalog number: 302019), and PE CD19 (BioLegend, catalog number: 302208). The selection of viability maker and conjugated antibodies is dependent on the flow cytometer used for analysis and its laser/filter configurations

21. RBC lysis buffer/fixation solution (10×) (BioLegend, catalog number: 422401)

22. Hanks balanced salt solution (HBSS) (Gibco, catalog number: 14025076)

23. DMSO (Thermo Scientific Chemicals, catalog number: J66650.AK)

24. QIAamp Viral RNA Mini kit (250) (QIAGEN, catalog number: 52906)

25. AgPath-ID^TM^ One-Step RT-PCR reagents (Thermo Scientific, catalog number: 4387391)

26. Forward primer (Integrated DNA Technologies, 5’-CGGGTTTATTACAGGGACA-3')

27. Reserve primer (Integrated DNA Technologies, 5’-C ACAATCATCACCTGCCAT-3')

28. ISCA probe (Integraated DNA Technologies, 5’-6FAM-AAAGGTGAAGGGGCAGTAGTAATACA-BHQ1-3’)

29. Tenofovir (TDF) (Gilead Sciences)

30. Emtricitabine (FTC) (API Sciences)

31. Dolutegravir (DTG) (Gilead Sciences)

32. Kleptose HPB (w/w) (Roquette, CAS number: 128446-35-5)

33. 4% Paraformaldehyde (PFA) solution (ChemCruz, catalog number: sc-281692)

34. Opti-MEM (Gibco, catalog number: 31985062)

35. FuGENE 6 transfection reagent (Promega, catalog number: E2691)

**Solutions**

1. D10 (see Recipes)

2. R10 (see Recipes)

3. R10-50 (see Recipes)

4. MACS buffer (see Recipes)

5. Freezing media (see Recipes)

6. Tenofovir (TDF), Emtricitabine (FTC), and Dolutegravir (DTG) antiretrovirals (see Recipes)

**Recipes**

**1. D10**

**Table:** 

Reagent	Final concentration	Quantity or Volume
DMEM	n/a	1,000 mL
FBS	10%	100 mL
HEPES	1%	10 mL
Penicillin/streptomycin	1%	10 mL

**2. R10**

**Table:** 

Reagent	Final concentration	Quantity or Volume
RPMI-1640	n/a	1,000 mL
FBS	10%	100 mL
HEPES	1%	10 mL
L-Glutamine	1%	10imL
Penicillin/streptomycin	1%	10 mL

**3. R10-50**

**Table:** 

Reagent	Final concentration	Quantity or Volume
RPMI-1640	n/a	1,000 mL
FBS	10%	100 mL
HEPES	1%	10 mL
L-Glutamine	1%	10imL
Penicillin/streptomycin	1%	10 mL
IL-2	50 units/mL	n/a

**4. MACS buffer**

**Table:** 

Reagent	Final concentration	Quantity or Volume
DPBS	n/a	1,000 mL
FBS	20%	20 mL
EDTA	2 mM	n/a

**5. Freezing media**

**Table:** 

Reagent	Final concentration	Quantity or Volume
FBS	90%	0.9 mL
DMSO	10%	0.1 mL

**6. Tenofovir (TDF), Emtricitabine (FTC), and Dolutegravir (DTG) antiretroviral (ARV) cocktail**

Prepare 15% Kleptose HPB (w/w) in water. Add 80 mL of 15% Kleptose to a volumetric flask and stir in 250 mg of DTG-free base. Stir and sonicate for 30 min or until soluble. Add 510 mg of TDF and 4,000 mg of FTC to the same flask and stir for 3 min. Fill to 100 mL with 15% Kleptose. Confirm the pH is 4.2, filter sterilize through a 0.22 μM filter, and store aliquots at -20 °C until ready for use.

**Table:** 

Reagent	Final concentration	Quantity or Volume
15% Kleptose HPB (w/w)	n/a	100 mL
TDF	5.1 mg/mL	510 mg
FTC	40.0 mg/mL	4,000 mg
DTG	2.5 mg/mL	250 mg

**Laboratory supplies**

1. Nunc T75 tissue culture flask (Thermo Scientific, catalog number: 156499)

2. 0.45 μm filter (Corning, catalog number: 431220)

3. 0.22 μm filter (Millipore Sigma, catalog number: SE1M179M6)

4. 96-well black/clear bottom plate, TC surface (Thermo Scientific, catalog number: 165305)

5. Nunc 15 mL conical sterile centrifuge tubes (Thermo Scientific, catalog number: 339650)

6. Nunc 50 mL conical sterile centrifuge tubes (Thermo Scientific, catalog number: 339652)

7. Alcohol prep pad (Mckensoon, catalog number: 191089)

8. Gauze pads 2 × 2 (Fisher Scientific, catalog number: 50-118-0367)

9. Surgical blade stainless steel No. 10 sterile (McKesson, catalog number: 1029067)

10. Microvette^®^ 100 blood collection tubes (potassium EDTA 100/pk) (Kent Scientific, catalog number: MCVT100-EDTA)

11. Kwik-Stop^®^ styptic powder with benzocaine (ARC International, catalog number: B000093HIR)

12. Cotton-tipped applicator swab stick, sterile, wood shaft (McKesson, catalog number: 24-106-2S)

13. Lo-Dose^TM^ U-100 syringes (28G needle) (BD Biosciences, catalog number: 329461)

14. MicroAmp fast optical 96-well reaction plate, 0.1 mL (Applied Biosystems, catalog number: 4346907)

15. MicroAmp optical adhesive strip (Applied Biosystems, catalog number: 4311971)

16. 1.1 mL microtubes (Thermo Scientific, catalog number: 15086)

## Equipment

1. CO_2_ incubator (Fisher Scientific, catalog number: 13-998-253)

2. Cell culture laminar flow hood, vacuum suction, temperature-controlled centrifuge, cell counter

3. SpectraMax i3x (Molecular Devices, catalog number: 10014-924) or equivalent

4. Mouse housing and handling facility

5. Heat lamp with base (Braintree Scientific, catalog number: HL-1B)

6. Rotating tail injector/restrainer (Braintree Scientific, catalog number: RTI)

7. EasySep^TM^ magnet (for cell isolation) (STEMCELL Technologies, catalog number: 18001)

8. Attune NxT flow cytometer (Invitrogen, catalog number: A24858) or other flow cytometer

9. QIAcube HT (Qiagen, catalog number: 9001896) (optional)

10. QuantStudio 7 Pro Real-Time PCR System (Applied Biosystems, catalog number: A43183) or equivalent

11. T-Flex^®^ Plus small animal handling glove (Lomir Biomedical Inc., catalog number: HG013G7)

## Software and datasets

1. FlowJo (version 10.10), requires a license

2. Design and Analysis 2 (DA2) software (version 2.8.0), free

## Procedure

*Note: All procedures conducted in mice must first have Institutional Animal Care and Use Committee approval (IACUC). Use of human PBMCs also requires Institutional Review Board Approval and/or Scientific Ethics Committee and participant informed consent.*

**A. Preparation of HIV stock**

*Note: This method utilizes HIV* ***_JRCSF_*** *for infection of mCD4+ T cells in mice; it is likely that other CCR5- and CXCR4-tropic HIV strains can be substituted, including autologous virus.*

1. Plate 7.5 × 10^6^ HEK293T cells in 20 mL of DMEM in a T75 flask to obtain a confluency of 80%–85% the next day. Incubate the cells at 37 °C with 5% CO_2_ overnight (ON).

2. Prepare transfection master mix by diluting 48 μL of Fugene6 dropwise in 600 µL of Opti-MEM and incubate for 5 min at room temperature (RT). Add the entire volume dropwise to a new tube containing 16 µg of full-length HIV JR-CSF plasmid (pYK-JRCSF) and incubate for 20 min at RT before transferring the mix dropwise to the cells. Incubate the cells at 37 °C with 5% CO_2_ ON.

3. Remove the media and add 20 mL of fresh D10 to the T75 flask. Incubate the cells at 37 °C with 5% CO_2_ ON.

4. Transfer the supernatant into a 50 mL Falcon tube and spin at 400× *g* for 10 min.

5. Filter the supernatants through a 0.45 μm filter into a new 50 mL Falcon tube.

6. Add PEG-it to the filtered supernatants in a 1:5 dilution and incubate at 4 °C ON (>12 h).

7. To pellet the virus particles, centrifuge the supernatants at 1,500× *g* at 4 °C for 30 min and aspirate the supernatant.

8. Resuspend the pellet in 20 mL of R10 and store aliquots of the virus at -80 °C until use.

**B. Determination of viral titer by TZM-bl assay**

*Note: This protocol was described in [15].*

1. Add 100 μL of D10 into the wells of a black 96-well plate.

2. Add 25 μL of HIV stock into the first column of wells and make a 5-fold dilution series of the virus.

3. To each well, add 1 × 10^4^ TZM-bl cells in 100 μL of D10 supplemented with DEAE dextran at a final concentration of 30 μg/mL.

4. Incubate the plate at 37 °C with 5% CO_2_ for 48 h.

5. Aspirate the supernatant and wash the cells in 200 μL of PBS per well.

6. Dilute 5× cell culture lysis reagent to 1× in distilled water and add 30 μL per well. Shake the plate for 15 min.

7. Add 50 μL of luciferase substrate to each well and incubate at RT for 2 min. Measure the luminescence using a SpectraMax i3x.

8. Determine the TCID50 of the virus using the TCID50 macros.

*Notes:*

*1. TCID50 macros can be downloaded from hcv.lanl.gov (* *https://hcv.lanl.gov/content/nab-reference-strains/html/TCID501.xls* ).

*2. mCD4+ T cells in mice are infected with 10,000 TCID50 HIV* ***_JRCSF_*** *in a total volume of 100 μL of R10 via intraperitoneal (IP) injection (see section H). Thus, the virus stock must have a titer of a minimum of 100,000 TCID50.*

**C. mCD4+ T-cell isolation and purity check**

1. Thaw cryopreserved PBMCs and resuspend in MACS buffer to a concentration of 5 × 10^7^ cells/mL.

*Note: Use of human PBMCs requires prior ethical approval by an institutional review board and participant informed consent.*

2. Isolate mCD4+ T cells by negative selection using EasySep^TM^ Human Memory CD4+ T-cell Enrichment kit according to the manufacturer’s protocol.

3. Resuspend the enriched mCD4+ T cells to a concentration of 2–5 × 10^6^ cells/mL in R10-50 and transfer the cells to an appropriately sized culture flask.

4. Rest the cells at 37 °C with 5% CO_2_ for a minimum of 5 h or ON before preparing the cells for engraftment.

5. During incubation, assess the purity of the enriched mCD4+ T cells by staining 50 μL of cell suspension for flow cytometric analysis using a viability marker and the following anti-human surface marker antibodies: CD3, CD4, CD8, CD45RO, CD56, CD14, CD16, and CD19.

**Critical**: The purity of the enriched mCD4+ T cells should be at least 95% to proceed with engraftments. If a purity of <95% is obtained, further purification should be performed. Depending on the source of contamination, perform a second round of negative CD4 enrichment or CD8 depletion. Optionally, a CD8 depletion can always be performed to minimize the risk of xenoreactive CD8+ T-cell proliferation.

*Note: See [14] and the Reagent section (no. 19) for a flow cytometry panel reference.*

**D. mCD4+ T-cell engraftment**

1. After incubation, transfer mCD4+ T cells to a 50 mL conical tube and centrifuge at 500× *g* for 5 min.

2. Resuspend in HBSS to a final concentration of 5–7 × 10^7 ^cells/mL and store on ice for a maximum of 1 h.

**Critical**: Prepare excess volume of cells to account for needle dead space.

3. Mix mCD4+ T cells by inversion, load 100 μL of the cell suspension into a 28G needle, and engraft cells via tail intravenous (IV) injection to each mouse.

**Caution:** Cells from PWH may contain replication-competent virus; thus, a needlestick injury carries a risk of HIV infection. A safety plan must be implemented to minimize the risk of exposure to personnel performing procedures involving the use of needles or any sharp instruments and to have emergency procedures in place in case of needlestick injury.

*Note: See General note 4 for considerations of mice age*.

**E. Tail nick blood collection and monitoring of mCD4+ T-cell engraftment by flow cytometry**

1. Up to once per week, starting 3 weeks after mCD4+ T-cell engraftment, collect up to 100 μL of blood into EDTA-coated tubes via a tail vein nick technique.

2. Centrifuge blood in the EDTA-coated tube at 5,000× *g* for 5 min to separate plasma.

3. Remove the plasma layer, transfer to an appropriately sized storage tube, and store at -80 °C before viral RNA extraction.

*Note: See section I for RNA extraction*.

4. Determine the amount of plasma volume transferred from the EDTA-coated tube and add an equivalent volume of DPBS to the EDTA-coated tube.

5. Mix blood and DPBS mixture and transfer 100 μL of this mixture into a 1.1 mL microtube.

6. Immediately stain blood for flow cytometric analysis with any desired anti-human antibodies and CountBright Absolute counting beads.

*Note: At a minimum, anti-human CD45, CD3, CD4, and CD8 should be included in the flow panel to quantify and enumerate CD4+ T cells and confirm no CD8+ T-cell contamination.*

7. After antibody stain, lyse red blood cells (RBC) with RBC lysis buffer by incubation at RT for 15 min in the dark according to the manufacturer’s instructions.

*Note: RBC lysis buffer is supplied in a 10× solution and must be diluted 10-fold in distilled water.*

8. Centrifuge the samples at 500× *g* for 5 min, aspirate supernatant, and resuspend cell pellet in PBS buffer.

9. Centrifuge the samples at 500× *g* for 5 min and aspirate supernatant. Fix cells by resuspending pellet with 200 μL of 4% PFA and incubate at RT for 10 min in the dark.

10. Centrifuge the samples at 500× *g* for 5 min, aspirate supernatant, and resuspend cell pellet into a single cell suspension in MACS buffer.

11. Analyze samples on Attune NxT flow cytometer and FlowJo software.

*Note: Analysis can be completed with any flow cytometer and compatible software.*

12. Monitor mCD4+ T-cell counts until levels exceed 50 mCD4+ T cells/µL. See Figure 1 for typical cell expansion.

*Note: For a successful infection, mCD4+ T-cell counts must reach a minimum of 10 cells/µL of peripheral blood before proceeding with the protocol. Exceeding the threshold of 50 mCD4+ T cells/µL is strongly recommended and typically requires 3–6 weeks post mCD4 + T-cell engraftment.*

**F. Cage rearrangement**

1. Rearrange mice into the desired number of groups to achieve an equal distribution of mCD4+ T-cell mean, median, and standard deviation between groups.

*Note: Extreme outliers including mice with mCD4+ T-cell counts below 10 cells/μL should be excluded.*

**G. mCD8+ T-cell isolation and purity check**

1. Three to six weeks after mCD4+ T-cell engraftment and cage rearrangement (see sections E and F), thaw cryopreserved PBMCs and resuspend in MACS buffer to a concentration of 5 × 10^7^ cells/mL.

2. Isolate memory CD8+ (mCD8+) T cells by negative selection using EasySep^TM^ Human Memory CD8+ T-cell Enrichment kit according to the manufacturer’s protocol.

3. Resuspend the enriched mCD8+ T cells to a concentration of 2 × 10^6^ cells/mL in R10-50 and transfer the cells to an appropriately sized culture flask.

4. Rest the cells at 37 °C with 5% CO_2_ for a minimum of 5 h or ON before preparing the cells for engraftment.

5. During incubation, assess the purity of the enriched mCD8+ T cells by flow cytometry.

**Critical:** The purity of the enriched mCD8+ T cells should be at least 95% to proceed with engraftments. If a purity of <95% is obtained, further purification should be performed. Depending on the source of contamination, perform a second round of negative CD8 enrichment.

*Note: The same anti-human antibody panel used in step C5 can be used for this purity assessment.*

**H. mCD8+ T-cell engraftment and in vivo infection**

1. After the incubation, transfer mCD8+ T cells to a 50 mL conical tube and centrifuge at 500× *g* for 5 min.

2. Resuspend in HBSS to a final concentration of 5–7 × 10^7^ cells/mL and store on ice for a maximum of 1 h.

**Critical:** Prepare excess volume of cells to account for needle dead space.

3. Thaw HIV**_JRCSF_** stock on ice and dilute to 10,000 TCID50 HIV**_JRCSF_** in 100 μL of R10 media. Store on ice for a maximum of 1 h.

**Critical:** Prepare excess volume of virus to account for needle dead space.

4. Mix mCD8+ T cells by inversion, load 100 μL of the cell suspension onto a 28G needle, and engraft cells via tail IV injection to each mouse.

**Caution:** Cells from PWH may contain replication-competent virus; thus, a needlestick injury carries a risk of HIV infection. A safety plan must be implemented to minimize the risk of exposure to personnel performing procedures involving the use of needles or any sharp instruments and to have emergency procedures in place in case of needlestick injury.

5. Mix HIV_JRCSF _by inversion, load 100 μL of HIV**_JRCSF_** into a 28G needle, and administer to the mouse via IP injection.

**Caution:** This method utilizes replication-competent HIV; thus, a needlestick injury carries a risk of HIV infection. A safety plan must be implemented to minimize the risk of exposure to personnel performing procedures involving the use of needles or any sharp instruments and to have emergency procedures in place in case of needlestick injury. In addition, personnel must wear needlestick-resistant gloves for procedures that require IP injection.

**I. Monitoring plasma HIV-1 RNA concentrations**

*Note: This protocol was described in Cillo et al. [16].*

1. Extract RNA from 30 μL of plasma using the QIAamp Viral RNA Mini kit according to the manufacturer’s protocol. Elute RNA in a volume of 80 μL of nuclease-free water and store extracted RNA at -80 °C.

*Note: If mid- to high-throughput RNA extraction is desired, the QIA cube HT system using the same consumables is available.*

2. Using the AgPath-ID One-Step RT-PCR kit, prepare a master mix with a total volume of 16.5 μL per reaction containing 12.5 μL of 2× buffer, 1 μL each of forward (10 μM, 5’- CGGGTTTATTACAGGGACA -3’) and reverse (10 μM, 5’- CACAATCATCACCTGCCAT -3’) primers [17], 1 μL of probe (6.25 μM, 5’-6FAM-AAAGGTGAAGGGGCAGTAGTAATACA-BHQ1-3’)[18], and 1 μL of N2 polymerase.

3. Add 8.5 μL of validated HIV RNA standards (1–10,000 copies/μL), 8.5 μL of extracted plasma RNA, or 8.5 μL of nuclease-free water for a total reaction volume of 25 μL.

4. Perform qPCR in duplicates using the QuantStudio 7 Pro Real-Time PCR system and Design and Analysis 2 (DA2) software (version 2.8.0) (or equivalent instrument and software). See Table 1 for cycling parameters.

5. Compare cycle threshold values with the validated HIV RNA standards run on each plate to determine plasma HIV RNA concentration (see Data Analysis).

*Note: See Troubleshooting 1.*

**Table 1.** Thermocycling conditions for the qPCR reaction

Step	Temp. (°C)	Duration	No. of cycles
cDNA synthesis	45	10 min	1
Initial denaturation	95	10 min	1
PCR	95	15 s	40
	60	1 min	

**J. Antiretroviral treatment**

1. Thaw the TDF/FTC/DTG mixture at 4 °C or RT.

*Note: TDF/FTC/DTG mixture can be kept at 4–8 °C for up to 1 week.*

2. Load 25 μL of ARV cocktail (see Recipes) into a 28G needle and administer daily to the mouse via subcutaneous (SC) injection. Dose at 5.1 mg/kg TDF, 40 mg/kg FTC, and 2.5 mg/kg DTG.

**Caution:** A safety plan must be implemented to minimize the risk of exposure to personnel performing procedures involving the use of needles or any sharp instruments and to have emergency procedures in place in case of needlestick injury. In addition, personnel must wear needlestick-resistant gloves for procedures that require SC injection.

*Note: Mice are typically treated with ARVs for 3 weeks to achieve undetectable plasma HIV-1 RNA concentrations. Low-level HIV-1 RNA is observed in a subset of mice, which typically returns to an undetectable concentration by the next sampling period. Increased mCD4+ T-cell counts during ARV treatment are often observed.*

**K. Tissue harvest**

1. At study termination, euthanize the mice and harvest organs per IACUC guidelines.

2. Transfer organs to prewarmed R10 media and process immediately to ensure high cell viability.

*Notes:*

*1. A majority of mCD4+ and mCD8+ T cells are recovered from the spleen, followed by the bone marrow, and sparingly from the lungs. Spleen and bone marrow cell viability is typically >90%. Lung cell viability is <30% due to cell death caused by CO_2_ euthanasia*.

*2. As described by Jackson Laboratories, lymphoid cell follicles in the spleen and cystic structures in the thymus are absent in NSG mice. There is also diminished cellularity of lymph nodes [19].*

3. Use cells immediately for flow cytometric analysis or other in vitro assays. Cells can also be cryopreserved in freezing media and stored in liquid nitrogen.

## Data analysis

To interpolate the cell count per microliters (mm^3^) of blood, 5 μL of CountBright counting beads are used per 100 μL of blood. Determine the expected number of beads per sample and normalize cell counts using the bead yield in each sample.

$$Cells/uL\;of\;blood\;=\frac{\left(CD4+or\;CD8+T-cell\;count\right)\;x\;expected\;bead\;count}{total\;bead\;count}\;\times \;1,000$$

To quantify plasma HIV RNA levels, utilize the absolute quantification using a standard curve method. Copies (Cp) per milliliter of plasma are quantified by interpolating the average quantification cycle (Cq) considering the volume of extracted RNA per reaction (8.5 µL), the total RNA elution volume (80 µL), and the volume of plasma imputed for the RNA extraction (30 µL) and scaled to Cp/mL.

$$HIV\;RNA\frac{copies}{mL}of\;blood\;=\frac{10^{\left(\;\frac{Average\;sample\;Cq-standard\;curve\;y-intercept}{standard\;curve\;slope}\right)}}{8.5\;\times 30}\times 80\times 1,000$$

## Validation of protocol

This protocol or parts of it has been used and validated in the following research article(s):

- McCann et al. [14]. A participant-derived xenograft model of HIV enables long-term evaluation of autologous immunotherapies. J Exp Med.
- Gramatica et al. [18]. The EZH2 inhibitor tazemetostat mitigates HIV immune evasion, reduces reservoir formation, and promotes durable CD8+ T-cell revitalization. bioRxiv.
  **Figure 1.**
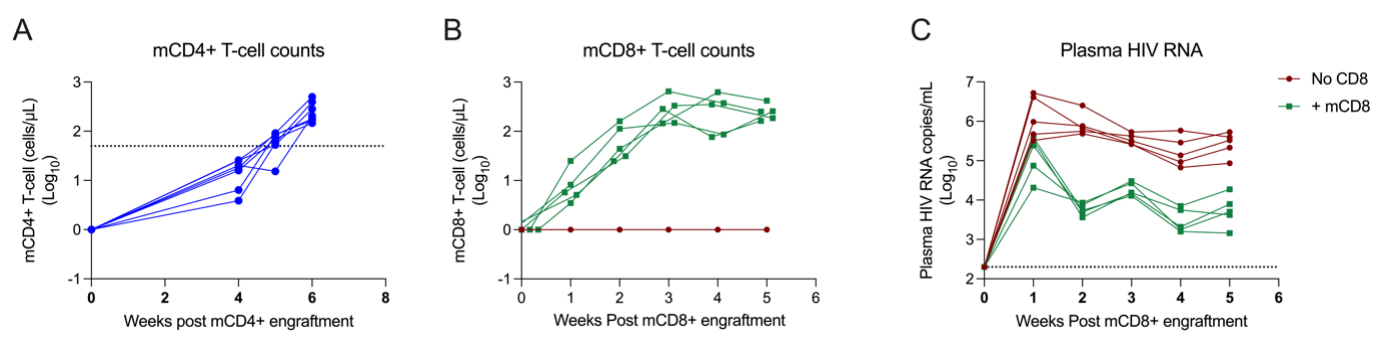
mCD4+ and mCD8+ T-cell expansion following tail IV engraftment. A. mCD4+ T-cell counts 6 weeks after mCD4+ T-cell engraftment. B. mCD8+ T-cell counts 5 weeks post mCD8+ T-cell engraftment. C. Plasma HIV-1 RNA concentrations 5 weeks post simultaneous mCD8+ T-cell engraftment and HIV infection.

## General notes and troubleshooting

**General notes**

1. GvHD is mild in the HIV PDX model primarily due to the use of memory T cells instead of all T-cell subsets. Some weight loss, hunching posture, and fur loss are observed. If weight loss exceeds 15% of initial body weight, diet gels can be used to mitigate further weight loss. GvHD is ameliorated after HIV**_JRCSF_** infection primarily due to the associated decrease in mCD4+ T-cell counts.

2. Cryopreserved isolated T cells: Although it is preferred to use freshly isolated mCD4+ and mCD8+ T cells for engraftments to maximize cell yields, previously isolated cryopreserved cells can be used.

3. Timeframe: The minimum time needed for this protocol is 6 weeks, assuming a minimum of 3 weeks for mCD4+ T-cell expansion and 3 weeks for all mice to reach detectable levels of plasma HIV RNA. The longest experiment conducted by our group exceeded one year from the time of mCD4+ T-cell engraftment. At longer experiment durations, T-cell counts will decrease in peripheral blood but will remain present in tissues (primarily the spleen).

4. Age of mice: The mice should be 6 weeks old or older to be mature enough before mCD4+ T-cell engraftment.

**Troubleshooting**

Problem 1: Detectable plasma HIV RNA before HIV infection.

Possible cause(s): Contamination in qPCR reaction or autologous virus rebound.

Solution(s): Ensure and maintain contamination-free spaces. Amplification in no-template control (NTC) wells is evidence of contamination. If the NTC wells have no amplification, and mCD4+ T cells from PWH were used for engraftment, autologous viral rebound is possible. In the HIV PDX model, autologous virus rebound is also correlated with a decrease in mCD4+ T-cell populations. At the first sampling time point after mCD4+ T-cell engraftment, quantify plasma HIV RNA. If autologous virus rebound is part of the experimental design, plasma collected from these HIV RNA-positive mice can be used to infect the remaining mice in the study.

Problem 2: Low yields of isolated mCD4+ T cells.

Possible cause(s): HIV infection is associated with the progressive decline of circulating CD4+ T cells in PWH.

Solution(s): With a small aliquot of PBMCs from the same donor and collection timepoint that will be used for engraftment, complete a mCD4+ T-cell isolation to determine the average mCD4+ T-cell yield. Use enough starting PBMCs to yield 5–7 × 10^6^ mCD4+ T cells per mouse. Alternatively, engraftment with fewer than 5 × 10^6^ mCD4+ T cells is possible but may result in a longer time for mCD4+ T-cell expansion to reach the desired level.
